# “The 3/3 Strategy”: A Successful Multifaceted Hospital Wide Hand Hygiene Intervention Based on WHO and Continuous Quality Improvement Methodology

**DOI:** 10.1371/journal.pone.0047200

**Published:** 2012-10-22

**Authors:** Gabriel Mestre, Cristina Berbel, Purificación Tortajada, Margarita Alarcia, Roser Coca, Gema Gallemi, Irene Garcia, Mari Mar Fernández, Mari Carmen Aguilar, José Antonio Martínez, Jesús Rodríguez-Baño

**Affiliations:** 1 Nosocomial Infection Control Unit, Delfos Medical Center, Barcelona, Catalonia, Spain; 2 Supervisor Nursing Department, Delfos Medical Center, Barcelona, Catalonia, Spain; 3 Infectious Diseases Unit, Hospital Clinic, Barcelona, Catalonia, Spain; 4 Infectious Diseases and Microbiology Unit, Universitary Hospital Virgen de Macarena, Seville, Spain; University of Ottawa, Canada

## Abstract

**Background:**

Only multifaceted hospital wide interventions have been successful in achieving sustained improvements in hand hygiene (HH) compliance.

**Methodology/Principal Findings:**

Pre-post intervention study of HH performance at baseline (October 2007– December 2009) and during intervention, which included two phases. Phase 1 (2010) included multimodal WHO approach. Phase 2 (2011) added Continuous Quality Improvement (CQI) tools and was based on: a) Increase of alcohol hand rub (AHR) solution placement (from 0.57 dispensers/bed to 1.56); b) Increase in frequency of audits (three days every three weeks: “3/3 strategy”); c) Implementation of a standardized register form of HH corrective actions; d) Statistical Process Control (SPC) as time series analysis methodology through appropriate control charts. During the intervention period we performed 819 scheduled direct observation audits which provided data from 11,714 HH opportunities. The most remarkable findings were: a) significant improvements in HH compliance with respect to baseline (25% mean increase); b) sustained high level (82%) of HH compliance during intervention; c) significant increase in AHRs consumption over time; c) significant decrease in the rate of healthcare-acquired MRSA; d) small but significant improvements in HH compliance when comparing phase 2 to phase 1 [79.5% (95% CI: 78.2–80.7) vs 84.6% (95% CI:83.8–85.4), p<0.05]; e) successful use of control charts to identify significant negative and positive deviations (special causes) related to the HH compliance process over time (“positive”: 90.1% as highest HH compliance coinciding with the “World hygiene day”; and “negative”:73.7% as lowest HH compliance coinciding with a statutory lay-off proceeding).

**Conclusions/Significance:**

CQI tools may be a key addition to WHO strategy to maintain a good HH performance over time. In addition, SPC has shown to be a powerful methodology to detect special causes in HH performance (positive and negative) and to help establishing adequate feedback to healthcare workers.

## Introduction

Healthcare-associated infections (HAI) occur in 5–10% of hospitalized patients during their hospital stay [1]. HAI is a major source of anxiety to patients, to the public and is very costly to health services [2]. Healthcare workers' hands are known to be the most common vehicle for the transmission of healthcare-associated pathogens [3]. The importance of hand hygiene (HH) in preventing HAIs is well sustained in evidence-base models [4], [5], and prospective studies [6], [7], [8], [9], [10]; also, HH promotion is included in all bundle interventions aimed to reduce HAIs [1].

Although adherence to appropriate HH practices is considered one of the cornerstones for HAI prevention [3], [4], [11], following HH guidelines in many healthcare facilities remains suboptimal [12], with median compliance rates below 50% reflecting a worrying gap between evidence and real practice. The promotion of effective measures to improve HH is among the five foremost goals of the WHO current worldwide Patient Safety Initiative. Furthermore, in the 2008 Patient Safety goals [13] the Joint Commission requires hospitals to comply with WHO and/or Centers for Disease Control and Prevention HH guidelines [14].

Only hospital wide interventions aimed to promote a cultural change have been successful in achieving sustained improvements in HH compliance leading to diminished HAI rates [6], [7], [8], [9], [10]. Furthermore, knowledge from cognitive, behavioural, and social theories [15], [16], [17], [18], [19], [20], [21] and the contribution from focus groups [17], [22] have been extremely useful to understand the complexity of our goal and to overcome potential barriers. Thus, the interdependence of individual factors, environmental constraints and institutional climate [23] should be considered in strategic planning and development of HH promotion.

The Statistical Process Control (SPC) was initially developed at Bell laboratories by Dr Walter Shewhart [24] in 1924 and subsequently promoted by leaders in the field of Continuous Quality Improvement (CQI) as Deming and Juran [25]. The application of quality control charts to epidemiology and infection control was first suggested in 1984 [26]. In the early 1990s the Joint Commission on Accreditation of Healthcare Organizations (JCAHO) promoted CQI philosophy to improve health care delivery. Finally, in 1998 JCAHO standards introduced the concept of Statistical Process Control (SPC) to measure process improvement. The application of SPC to infection control is relatively new [27], [28], [29] and it requires the analysis of data through different types of control charts [25], [30], [31], [32], [33].

We undertook a 2 phase multifaceted hospital-wide HH intervention based on the multimodal WHO approach [34], [35] and CQI philosophy over 2 years, focusing on achieving a sustained HH cultural change in our institution. The objective of this study was to evaluate the impact and sustainability of this approach on HH compliance over time.

## Methods

The ORION statement for transparent reporting of intervention studies concerning healthcare-acquired infections was followed [36].

### Setting

Delfos Medical Center is a private 200-bed hospital with teaching nursing activity, with about 12,000 admissions and 50,000 patient-days each year. Almost 90% of the rooms are single. There are eight medical-surgical wards and a polyvalent intensive care unit (ICU) with 11 beds attending nearly 500 patients each year. A Nosocomial Infection Control Unit (NICU) was created in 2002 as part of the Infection Committee, which is formed by a full-time specialist in epidemiology and infectious diseases and by an infection control nurse.

### Study Design

We developed a “pre-post intervention” study through statistical comparison of HH performance at baseline and the two intervention phases. Furthermore, we performed prospective time series analysis through statistical process control (SPC) on HH during phase 2, alcohol hand rub solution (AHRs) consumption, and rate of healthcare-acquired MRSA colonization or infection (as detected by means of clinical samples only).The Ethics Committee from Delfos Medical Center approved conduct of the research without explicit consent from the participants because the management of our patients was not affected by the study.

### Interventions

The pre-intervention period (March 2007–December 2009) and the main characteristics of our 2-phase multifaceted hospital-wide intervention on HH, phase 1 from January throughout December 2010 and phase 2 from January throughout December 2011 are shown in table 1.

**Table 1:** Main characteristics of a 2 phase multifaceted hospital-wide hand hygiene intervention, Delfos Medical Center (2010–2011).

Periods and data	Description
**Preintervention period (March 2007–December 2009)**	Promotion of hand hygiene (HH) was performed but it was neither structured nor sustained on time. A limited HH campaign based on staff education, reminders (March 2007–October 2007) followed by limited six-month HH audit by direct observations (October 2007–April 2008) over a week (basal, and on month 3 and 6) was conducted. The alcohol hand rub solution (AHRs) was changed on June 2008 (Sterillium® gel, Bode Chemie, Hamburg, Germany); at this point, AHRs dispensers were located outside each room (corridor) and in the nursing carts. Isolation practices and HH promotion was reinforced during pandemic H1N1 threat (June2009-September 2009).

Hospital Wide Intervention	Phase 1 (January 2010–December 2010)	Phase 2 (January 2011–December 2011)
**Epidemiological context**	Catalonian Regional Campaign promoted by the “Alliance for Patient Safety” supported by WHO educational resources.	Continuous Quality Improvement (CQI) Promotion locally developed by Infection Control Unit and Supervisor's Nursing Department.
**1, Promotion of easy** **access to hand-rub** **solutions at points** **of care**	AHRs were placed at all bedsides on high risk areas (Emergency Department and Intensive Care Unit). At this point the ratio AHRs dispensers/bed was 0.57 (123/217).	AHRs were placed at all patient beds in conventional wards while maintaining those at corridors. At this point the ratio AHRs dispensers/bed was 1.56 (340/217).
**2. Staff education**	Theoretical and practical workshop was conducted directed to all HCWs categories (15 standardized slide presentations), accompanied by practical sessions encouraging good HH technique.	These actions was maintained without changes.
**3. Reminders (standard posters and** **lefts)**	Posters and handouts were donated by the promoters of subnational campaigns and were displayed in strategic areas previously identified by visiting the wards. Location criteria were maximal visibility during daily work and during transit within the hospitals. Posters were replaced monthly.	These actions were maintained without changes
**4. Audit**	A HH monitor team (HHMT) was created on March 2010 and included eight HCWs related to Infection Control Unit and Supervisor Nursing Department. Direct observations auditing was performed over three weeks (on June 2010) and two weeks (on October 2010). Thus, 2 evaluation periods and 25 days of monitoring were scheduled.	The HHMT and the methodology of observation procedure was maintained, but the periodicity of audits was changed as follows: Audits were performed during 3 randomized days every 3 weeks (“3/3 strategy”). Thus, 17 evaluation periods and 51 days of monitoring were conducted.
**5. Feedback**	Tables and bar graphs through were shown through informal interactive sessions on every ward at the end of evaluation period. Data were introduced in a centralized computer system for benchmarking.	Regularly bimonthly feedback using control charts (Statistical Process Control) on every ward at institutional and individual level were provided.
**6. Safety institutional** **climate**	Institutional Commitment by administrative and nursing director	This support was maintained during this period
**7. Proactive corrective actions**	Not performed	Corrective actions were registered in a specific form. Modification of incorrect HH habits, clarification of doubts and positive reinforcement were conducted.

In summary, phase 1 was based on the WHO hand hygiene multimodal (five steps) intervention approach (table 1), a standardized framework [34], [35] for training observers, performance of surveys and training of HCWs. Phase 2 was developed following the continuous quality improvement philosophy [32], [33].The main interventions added during phase II as regards phase I (table 1) were: a) increase of AHR dispensers placement (from 0.57 dispensers/bed to 1.56); b) increase of frequency audits (from 25 days to 51 days and audits were dispersed more evenly over time [2 vs 17 evaluation periods]); c) feedback was more standardized and statistical control graphs were shown to health care workers in a bimonthly fashion; and d) implementation of a standardized process for proactive corrective actions.

A hand hygiene monitor team (HHMT) was created on March 2010 and included eight HCWs. The team attended a theoretical and practical workshop following the WHO video methodology. The HHMT achieved a median theoretical correct responses rates of 93.4% (95% CI: 90.4–96.4%) after the WHO-recommended evaluation. Following WHO recommendations [35] four main professional categories were defined (assistant nurses, nurses, physicians, and “others” –including transport, laboratory and radiology technicians-) and 3 areas were defined (ICU, Emergency Department (ED) and medical-surgical wards). Observations were conducted at prespecified periods. Due to logistical reasons the weekends and night shifts were excluded. On each audit, all wards were monitored on the same day during 30 minutes except for ICU and ED where two different observations by two different HHMT members were planned. HCWs were informed about the observation schedule in advance. The observers were as unobtrusive as possible. The inter-observed variability [6] was also checked during audits, being the infection control nurse the reference with respect to all other auditors. The concordance was high for all variables among all HHMT members (mean kappa values  = 0.9; range = 0.85–0.91).

Finally, during the phase 2 of the intervention (2011), proactive corrective actions were also performed at the end of each observation period if deemed necessary by the auditor. This approach allowed us to clarify doubts of our HCWs concerning HH practices and to detect incorrect HH habits (meaning repetitive incorrect actions related to HH). In addition, an interactive and positive education approach without any punitive consequences was fostered. Corrective actions were registered in a specific form.

### Outcomes variables

The primary outcome was HH compliance calculated by dividing the number of HH episodes by the number of potential opportunities. The data was stratified by type of indications, working areas and professional category. Our retrospective control data included three sessions of HH audits performed over a week in October 2007, January 2008 and April 2008.These audits were performed following a similar procedure as that used during the intervention period (with the exception that the moment “after touching surroundings” was not evaluated) and were conducted also by nosocomial infection control and nursing supervisors' staff.

Secondary outcome variables were bimonthly AHRs consumption (in litres per 1,000 patient-days in each ward as provided by the Pharmacy account system) and the bimonthly healthcare-acquired colonisation/infection due to methicillin-resistant *Staphylococcus aureus* (MRSA) measured as the number of new cases per 1,000 patient-days identified from clinical, non-screening specimens as described previously [37]. Conventional microbiological procedures were used to identify MRSA isolates. Cases were identified from the infection control reports through total chart review. For MRSA rates, the preintervention period was the 2007–2009 period.

### Data analysis

Data were aggregated for the pre-intervention period, phase 1 intervention period and phase 2 intervention period. Differences in HH compliance at the different periods were analysed using χ^2^ tests for trends using Microsoft Windows SPSS (Statistical Package for the Social Sciences, 15.0). Also, time series analysis by Statistical Process Control (SPC) was performed by Minitab statistical software (Minitab®).

**Figure 1:**
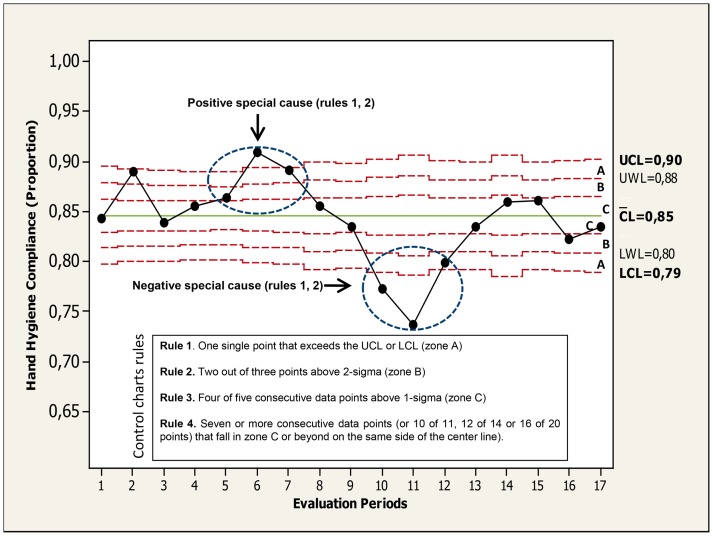
Binomial control chart (statistical overall hand hygiene compliance process control during phase 2). Audits were conducted during three randomized days every three weeks accounting for 17 evaluation periods on 2011. Two set of points are highlighted (circles) and the rules (“special causes”) are shown. Three zones (C, B, A) that emanate outward from the center line (CL) are labeled (often referred as “sigma limits”): zone C (from CL to +/− 1σ limit); zone B (from +/−1σ to +/− 2σ, whose limits are also known as “warning limits” [WL]), and zone A (from +/− 2σ to +/− 3σ [Upper control limit (UCL) and lower control limit (LCL) respectively].

The Statistical Process Control (SPC) approach [38] is based on learning through data and is sustained in the theory of variation. The variability of event rates (so-called “process” in chart terminology) over time can be classified as either “natural” or “unnatural”. Natural variability (also known as “common cause” or “inherent variation” in chart terminology) is defined as the systemic or random variation inherent in the process itself. On the other hand, observations with very few probabilities of occurrence based on the regular process are known as “special causes” (also known as non-systemic or unnatural variability) which could be related to fundamental changes in the process or environment. Special causes should be investigated, either in order to control it (negative special cause) or to incorporate it (positive special cause).Three horizontal lines are plotted on the chart referred as the center line (CL), upper control limit (UCL) and lower control limit (LCL). The statistical significance of changes is supported by mathematical rules that indicate when the data are not representing a random occurrence. The rules on chart performance have been widely described previously [25], [30], [31], [32], [33], [38], [39], [40]. A brief explanation of this rules are shown at the legend of figure 1. Finally, the mathematical approach is sustained on type of variable data. Briefly, P charts (binomial distribution) were constructed to plot the statistical control of HH compliance rate process during phase 2, U charts (Poisson distribution) were constructed to plot time series of AHRs consumption process (litres per 1,000 patient-days). Lastly, Poisson Exponential Weighted Moving Average (PEWMA) control charts were constructed to plot time series of healthcare-acquired MRSA infection/colonization rates process. These data were adjusted by patient-days.

For more related to control charts see text S1 (supporting information file).

## Results

During two years (2010–2011), 819 scheduled audit sessions were performed (277 in 2010 or phase 1 vs. 542 in 2011 or phase 2) which produced data for 11,714 HH opportunities (4,095 in 2010 vs. 7,619 in 2011). A median of 13 opportunities per audit sessions were recorded (range: 0–42) with no differences between intervention phase 1 and 2. Overall, time spent on auditing was 409.5 h (138.5 h in 2010 vs. 271 h in 2011). The HHMT dedicated an equivalent of 0.19 full working time/year (including 85 h/year related to analysis and interpretation of data).

Significant increase in HH compliance in the intervention periods was shown among all HH moments, HCWs, and working areas (table 2).The mean increase in HH compliance (intervention period vs preintervention period) was 25 percentage points (95% CI: 23.5–26.7; P<0001). During both intervention phases the patterns of HH compliance were similar: it was better in conventional wards than in ICU and ED, in nurses and assistant nurses than in physicians and others, and “after patient contact” than “before patient contact”.

**Table 2:** Hand hygiene compliance at preintervention period (t0), phase 1 intervention (t1) and phase 2 intervention (t2).

Variable	to	t1	t2	X^2^ for trend (p)
	March 2007– December 2009	January 2010– December 2010	January 2011– December 2011	
**No of observations**	3,881	4,095	7,619	
**Overall compliance, % (95% CI)**	57 (55.9–59.0)	78 (79.4–80.7)	84 (83.8–85.4)	<.0001
**Adherence to the 5 WHO HH moments**				
1. Before touching a patient				
No. of observations	1,281	1,681	2,736	
Compliance, % (95% CI)	43 (40.6–46.0)	76 (74.2–78.3)	82 (80.6–83.6)	<.0001
2. Before clean/aseptic procedure				
No. of observations	469	454	789	
Compliance, % (95% CI)	60 (55.7–64.6)	71 (66.9–75.3)	74 (71.3–77.7)	<.0001
3. After body fluid exposure risk				
No. of observations	567	315	661	
Compliance, % (95% CI)	73 (70.3–77.5)	82 (78.1–86.4)	83 (80.3–86.1)	<.0001
4. After touching a patient				
No. of observations	1,564	1,358	2,917	
Compliance, % (95% CI)	62 (59.9–64.7)	84 (82.7–86.5)	91 (90.1–92.2)	<.0001
5. After touching patient surroundings*				
No. of observations	NE	449	956	
Compliance, % (95% CI)	NE	95 (92.5–97.2)	77 (74.7–80.1)	
**HH adherence by HCW category**				
1. Nursing				
No. of observations	1,449	1,930	3,772	
Compliance, % (95% CI)	68 (65.6–70.4)	84 (82.2–85.6)	89 (87.5–89.6)	<.0001
2. Nursing assistants				
No. of observations	1,029	1,162	2,194	
Compliance, % (95% CI)	69 (66.3–71.9)	88 (89.6–91.4)	91 (90.1–92.3)	<.0001
3. Physicians				
No. of observations	724	662	1,123	
Compliance, % (95% CI)	48 (44.0–51.3)	60 (56.1–63.6)	63 (60.7–66.3)	<.0001
4. Others				
No. of observations	679	341	530	
Compliance, % (95% CI)	27 (24.3–31.05)	58 (52.8–63.3)	71 (67.7–75.4)	<.0001
**HH adherence by working area**				
1. Medical-Surgical Wards				
No. of observations	2,532	2,504	4,358	
Compliance, % (95% CI)	57 (55.1–58.9)	89 (88.3–90.7)	88 (87.1–89.0)	<.0001
2. Intensive Care Unit				
No. of observations	520	879	1,749	
Compliance, % (95% CI)	70 (65.9–73.6)	73 (70.1–75.9)	85 (82.9–86.4)	<.0001
3. Emergency Department				
No. of observations	829	712	1,512	
Compliance, % (95% CI)	51 (47.7–54.5)	52 (48.6–55.9)	74 (72.3–76.7)	<.0001

* Abreviations: NE, not evaluated.

When HH compliance was compared during phases 1 and 2 (table 2) significant differences were observed in overall HH compliance [78% (95% CI: 79.4–80.7) in phase 1 vs. 84% (95% CI: 83.8–85.4) in phase 2 (p<0.05)]. Furthermore, significant improvement was noted regarding before and after patient contact, in the ICU and ED (the latter being particularly relevant) and among nursing staff and radiology technicians. In terms of medical specialities (table 3) clinicians were significantly more compliant than surgeons. Notably, students, irrespective of their health care category, showed a significantly better compliance than its respective HCW category. Considering the number of opportunities per hour, as a proxy of index activity, the ICU (38.21 per hour) and nurses and assistant nurses (13.93 and 10.06 per hour, respectively) registered the highest figures.

**Table 3:** Main epidemiological characteristics of the two Intervention phases.

Variable	T1 (January 2010–December 2010)	T2 (January 2011–December 2011)
**Hand rub alcohol dispensers/beds (ratio)**	0.57 (123/217)	1.56 (340/217)
**Direct observation sessions performed** * **(n)**	277	542
**Opportunities for HH by session (median, IQR)**	14 (8–21)	13 (9–19)
**Overall time observation (hours)**	138.5	271
**Hand hygiene performance (%)**		
Alcohol	70.8	76.2
Soap	8.3	7.8
Alcohol & Soap	0.5	0.6
Not performed (not wearing gloves)	14.2	11.6
Not performed (wearing gloves)	6.3	3.8
**HCWs observed by session (average, SD)**		
Nurses	1.94 (0.9)	1.87 (0.9)
Assistant Nurses	1.73 (1.1)	1.73 (0.9)
Physicians	1.02 (1.1)	0.97 (1.1)
Others	0.61 (0.9)	0.52 (0.8)
**Hand hygiene opportunities/hour**		
Nurses	13.9	13.9
Assistant Nurses	10.1	9.8
Physicians	4.77	4.14
Others	2.46	1.95
**HH adherence by HCW subcategories**		
**Nursing**		
Nursing Staff		
N	1,803	3,347
Compliance, % (95% CI)	83 (81.3–84.8)	88 (87.5–89.6)
Student nurses		
N	127	425
Compliance, % (95% CI)	96 (92.6–99.4)	91.5 (88.9–94.2)
**Nursing assistants**		
Nursing assistants staff		
N	1,062	2,006
Compliance, % (95% CI)	89 (87.3–91.1)	91(89.9–92.4)
Student nursing assistants		
N	100	188
Compliance, % (95% CI)	95 (90.7–99.3)	92 (88.2–95.6)
**Physicians**		
Clinicians		
N	374	625
Compliance, % (95% CI)	72 (67.9–76.9)	69 (65.8–73.1)
Surgeons		
N	252	343
Compliance, % (95% CI)	37 (30.9–42.9)	46 (40.1–51.1)
Medicine students		
N	30	141
Compliance, % (95% CI)	93 (84.4–99.9)	84 (77.6–89.8)
**Others**		
Orderlies		
N	226	317
Compliance, % (95% CI)	65 (58.8–71.3)	72 (66.7–76.6)
Laboratory technicians		
N	50	129
Compliance, % (95% CI)	74 (61.8–86.2)	69 (61.1–76.9)
Radiology technicians		
N	65	84
Compliance, % (95% CI)	21 (11.5–31.5)	75 (65.7–84.3)

* All wards were monitored the same day for a 30 minute session except for Intensive Care Unit and Emergency Department where two different observations by two different hand hygiene monitor team (HHMT) members were planned.

**Figure 2:**
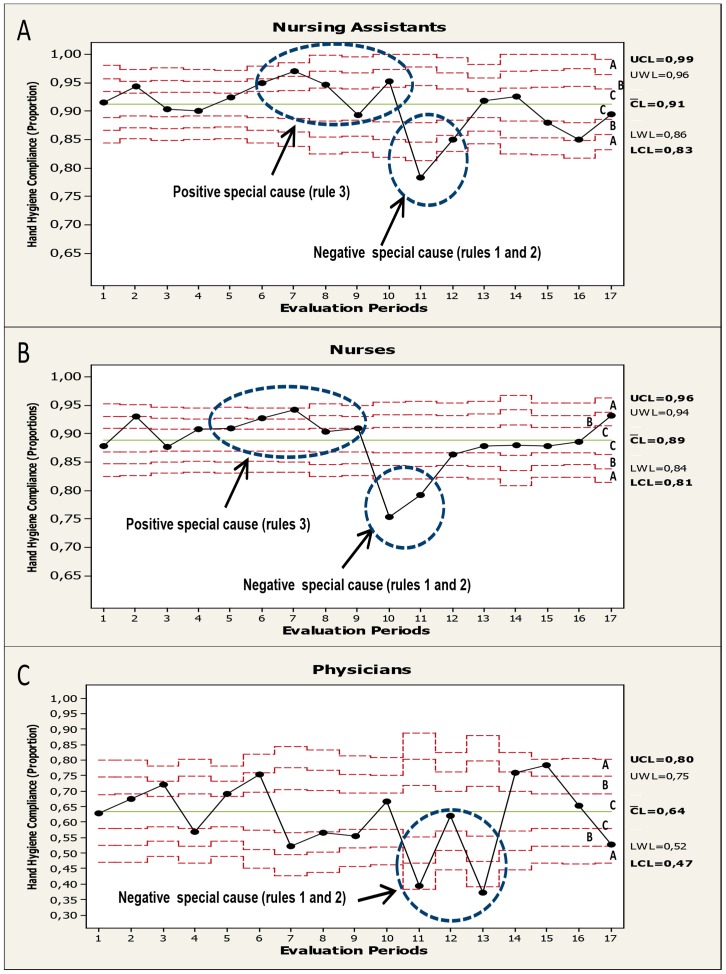
Binomial control chart (statistical hand hygiene compliance process control during phase 2 according HCW categories). A: nursing assistants. B: nurses. C: physicians. Sets of points are highlighted (circles) and the rules (special causes) are shown. Three zones (C, B, A) that emanate outward from the center line (CL) are labeled (often referred as “sigma limits”). See legend in Figure 1 for control charts rules explanation.

**Figure 3:**
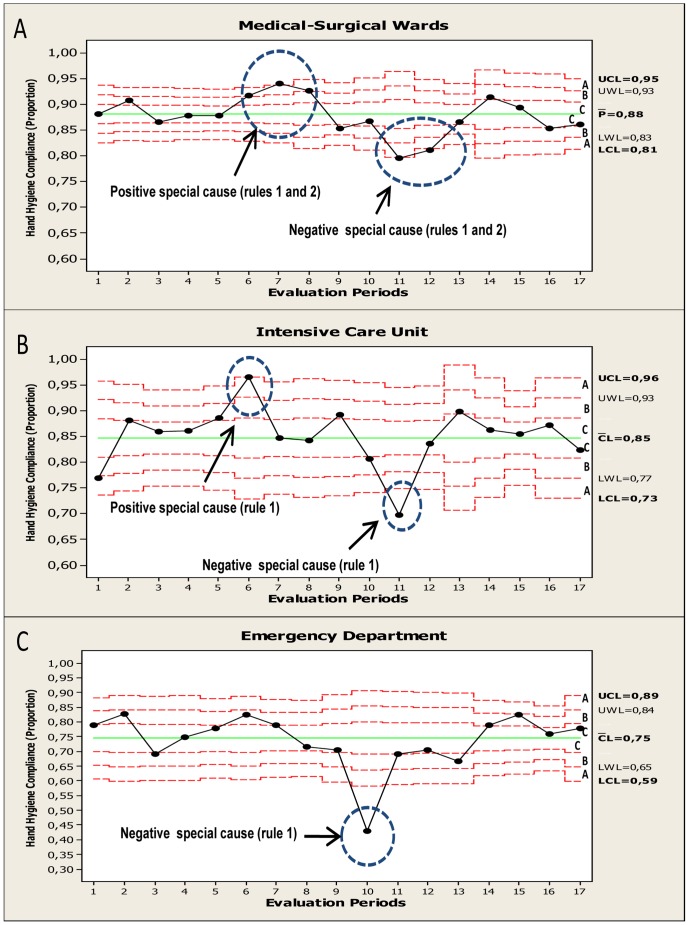
Binomial control chart (statistical hand hygiene compliance process control during phase 2 according working areas). A: medical-surgical wards. B: intensive care unit. C: emergency department. Sets of points are highlighted (circles) and the rules (special causes) are shown. Three zones (C, B, A) that emanate outward from the center line (CL) are labeled (often referred as “sigma limits”). See legend in Figure 1 for control charts rules explanation.

The Statistical Process Control (time series) of HH compliance process during phase 2 (2011) are shown in figure 1 (overall data); figure 2 (stratified by main HCWs categories) and; figure 3 (related to working area). Overall, the HH compliance process in phase 2 showed a mean compliance of 85% showing in certain periods a pattern of “non-random” variability (special causes).Two different types of “special causes” were noted: (1) A positive special cause (90.1% compliance) in the sixth evaluation period (during 4^th^, 5^th^, and 6^th^ of May 2011) and was coincident with “the World Hygiene Day”. (2) Negative special causes (lower value: 73.7% compliance) was observed in the 10th and 11th evaluation periods (during 26^th^,27^th^, 29^th^ of July and 16^th^, 18^th^ and 19^th^ of August, respectively) and affected nearly all HCWs categories (figure 2), working areas (figure 3), and type of indication (data not shown).These evaluations coincided with the statutory lay-off proceeding that took place in our Center at that time.

**Figure 4:**
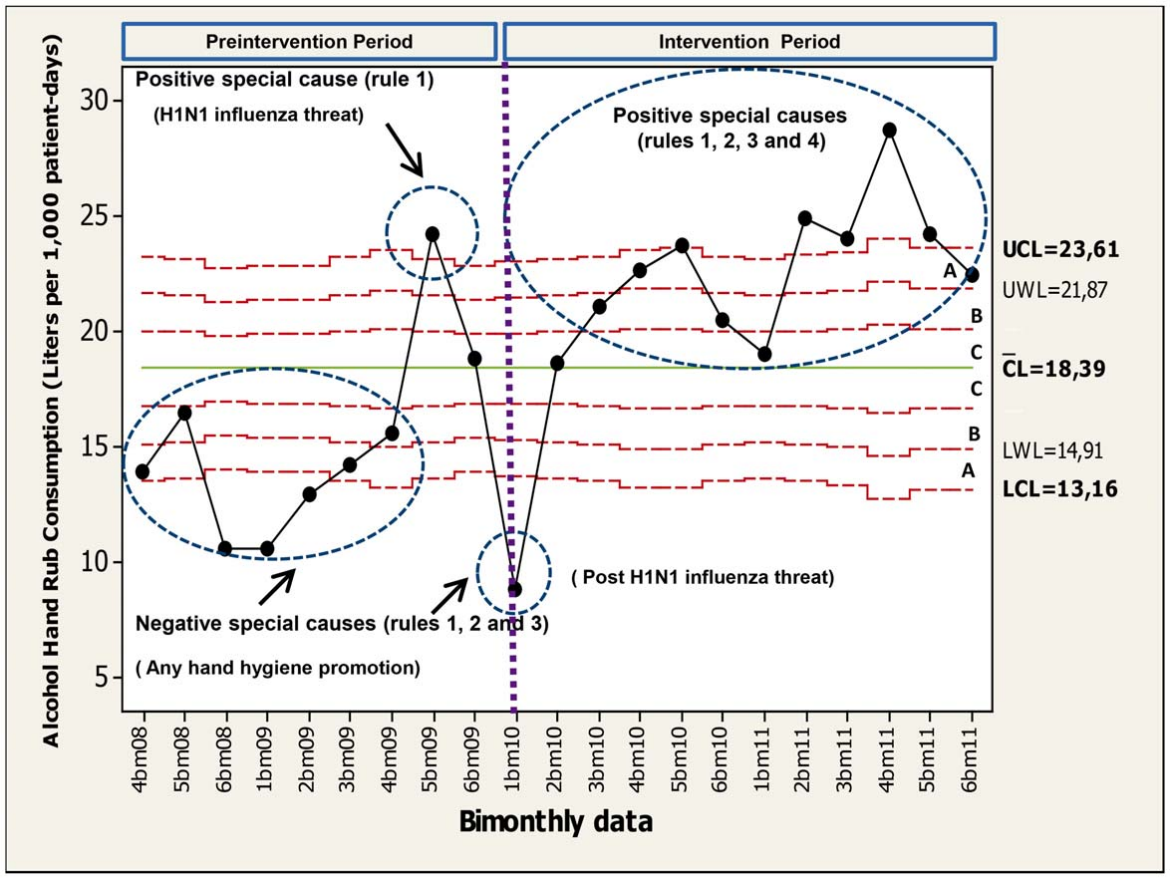
Poisson control chart (statistical overall alcohol hand rub consumption [liters/1,000 patient-days] process control). Data are shown in a bimonthly fashion from 4b m08 (July–August 2008) to 6bm11 (November–December 2011). Set of points are highlighted (circles) and the rules (special causes) are shown. See legend in figure 1 for control charts rules explanation.

Statistical control related to “bimonthly AHRs consumption process” is shown in figure 4. From July 2008 until December 2011, a 172% increase in the expenditure was noted achieving levels above 22 L/1,000 patient-days during 2011. Negative and positive special causes were noted in the 2008–2009 period and their probable aetiologies are shown. At the end of 2010 and 2011, numerous “positive special causes” were noted and a clear change of the process of “AHR consumption” was achieved.

**Figure 5:**
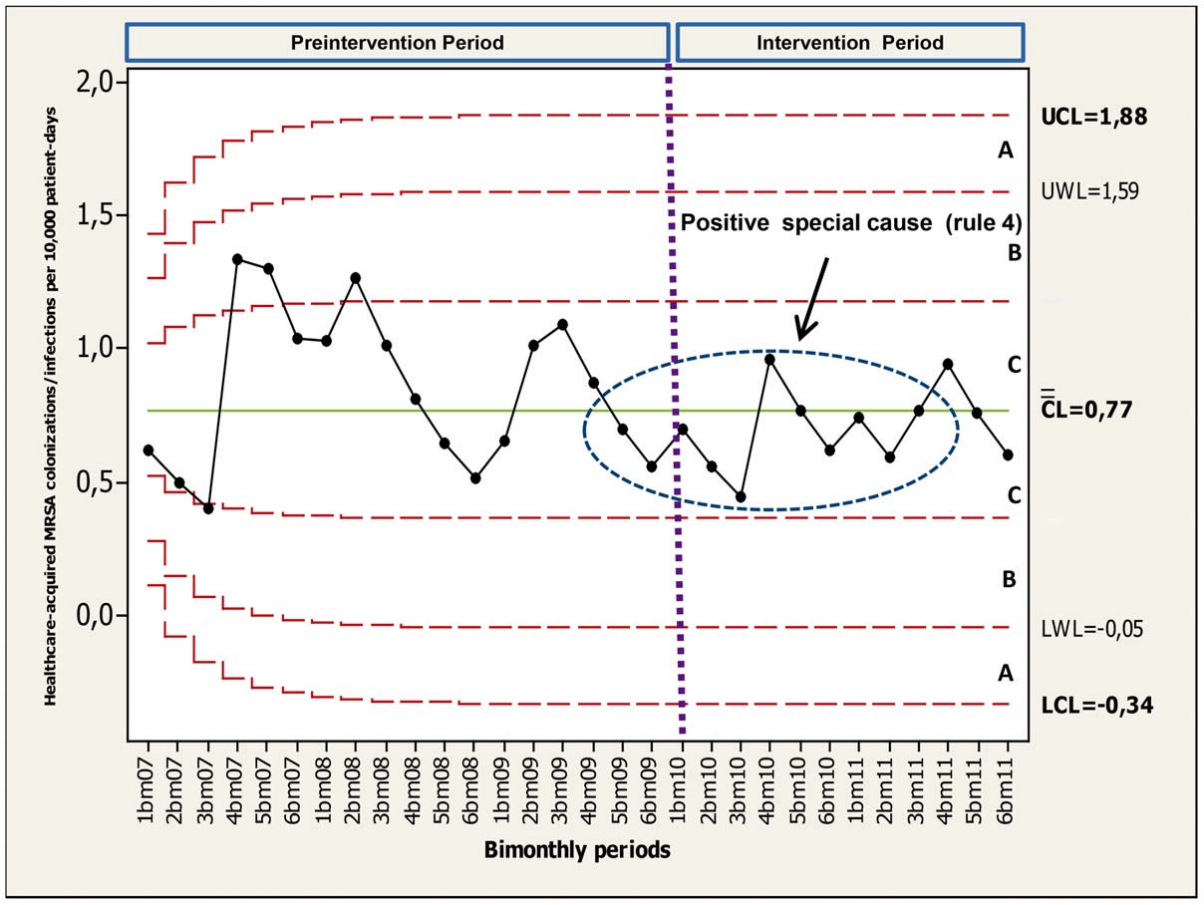
Poisson Exponential Weighted Moving Average control chart (statistical healthcare-acquired MRSA colonization/infection process control). Data are shown in cases per 10,000 patient-days (2007–2011 period). A set of points is highlighted (circles) and the rules (special causes) are shown. See legend in figure 1 for control charts rules explanation.

Time series analysis of “healthcare-acquired MRSA colonization/infections rates” process during the 2007–2011 period is illustrated through a Poisson Exponential Weighted Moving Average (PEWMA) control chart (see figure 5). This chart shows a low incidence rate over time (median of 0.77 per 10,000 patient-days) achieving a small but significant decrease in healthcare-acquired MRSA colonization/infections rates during the intervention period according to the rule that at least 10 out of 11 consecutive data points fall in zone C or beyond on the same side of the center line (referred as rule 4 in Figure 5).

During phase 2, up to 42 corrective actions were performed. Overall, 57% (n = 24) were aimed to discuss incorrect HH habits (repetitive incorrect actions related to HH), 33% (n = 14) were related to clarify doubts concerning HH practices and 16% (n = 4) were done to discuss missed specific HH opportunities (i.e.: no HH performance before aseptic technique). Main incorrect HH habits could be grouped in the following categories: a) wearing watches or jewels; b) fail to consider measuring blood pressure as a pre-patient opportunity for HH; c) missing HH opportunities when performing capillary blood glucose determinations; d) not performing HH “after touching patient surroundings” ; e) incorrect HH technique (according to WHO standardized HH technique); f) use of gloves instead of hand hygiene; and g) wearing gloves outside the room without justification.

Potential confounders of the putative effect of our intervention such as change in the case-mix (considering age, gender, length of hospital stay and weighted diagnoses-related group), did not changed over time (data not shown). As regards to overall antibiotic, and specifically fluoroquinolone consumption (DDD per 100 patient-days), there was a significant increase during the intervention period (overall consumption: 75.5 [95% CI: 75.3–75.6] vs 68.9 [95% CI: 68.7–69.1]; p<0.05; fluorquinolones consumption;17.0 [95% CI: 16.9–17.1] vs 16.4 [95% CI:16.3–16.5); p<0.05; intervention period vs preintervention period, respectively).

## Discussion

### Overview

The most remarkable findings of our strategy were: a) a significant improvement in HH compliance with respect to baseline (a 25 percentage point increase in the mean during the intervention period [2010–2011] with respect to the preintervention period [2007–2008]); b) a sustained high level (82%) of HH compliance during the intervention period; c) a significant increase in AHR consumption over time, with consistently significant rises in Phase 2; d) a significant decrease in healthcare-acquired MRSA infection/colonization coinciding with implementation of interventions; e) a small but significant improvement in HH compliance when comparing Phase 2 to Phase1 (particularly in the emergency department); and f) successful use of control charts to identify significant negative and positive deviations (special causes) in HH compliance over time.

### Main limitations and strengths

As potential limitations, this study describes a quality improvement project and we cannot ruled out that other unmeasured factors or potential confounders may have influenced the results. However, there were no changes in terms of patient characteristics (age, gender, length of hospital stay and DRGs) or infection control practices during the evaluation period and no outbreaks were detected. Second, this study is limited by its quasi-experimental design. Randomisation of the intervention was not feasible since it was performed in a single center and because its design was originally programmed for hospital-wide implementation. Third, a Hawthorne effect [41], [42], [43] may have occurred due to the fact that HCWs were aware of being observed. Fourth, we consider unlikely that a systematic change in the way clinicians ordered culture tests may have influenced the results of MRSA rates. Finally, our study was performed only in one centre with specific features.

The potential strengths of our study were the unusual large size of HH opportunities observed [12], and the novel use of CQI philosophy in our multimodal HH intervention (phase 2), highlighting the utility of Statistical Process Control (capable to detect either positive or negative “special causes”), immediate feedback to our HCWs and implementation of a standardized proactive corrective form that helped to gauge the extent of our intervention. To our knowledge, these aspects have not been previously analysed in detail.

### Comparison with other studies

Phase 1 intervention (2010) share key components with other successful hospital-wide WHO strategy based interventions [6], [7], [8], [9], [10] and included the five progressive steps such as administrative support and multidisciplinary approach, promotion of easy access to alcohol hand-rub solutions in points of care (AHR at bedside) educational interventions, strategically placed reminders, audits by direct observation and feedback on performance.

Phase 2 strategy (2011) added some particularities such as a continuous scheduled assessment of HH process and the application of a Statistical Process Control methodology. The use of brief monitoring audits (half an hour) maintained over time (three randomized days every three weeks) was shown a successful approach. In this regard some considerations should be taken into account: a) the methodology was by itself an improvement tool since it acted as a continuous reminder of the expected behaviour [17] from our HCWs and interacted with the subjective norm (a person's perception of pressure from peers and other social groups); b) it was an ideal scenario to encourage better performance, clarify doubts and modify “incorrect HH habits” in real time. This fact is shown by the 42 corrections made during phase 2 intervention. The immediate and individual feedback [44], [45] has been a key point in influencing HCWs performance; c) auditors can identify barriers to compliance and seek local solutions [46]; d) it is currently the only method that can detect all types of HH opportunities; and e) it is the only strategy that can provide detailed information about HH techniques.

Recently a successful HH program in which a key component was a continuous HH monitoring and feedback has been published [21]. Altogether, both strategies reinforces that frequent feedback is linked to improvement in healthcare quality [45].

Some drawbacks of HH direct observation audit have been identified [41], [47] mainly focusing in two aspects. First, it has been argued that it is labour intensive, time consuming and therefore expensive. This fact did not apply to our centre since it only represented a 0.19% of supervisor's nursing time (overall) and 15% of NICU dedication. Besides, some indirect data from previous studies [2], [10], [48] reinforces the cost/benefit of HH interventions. Second, it has been suggested that in order to ensure the quality of the audit process it is necessary to train and monitor the auditor regularly. In our case, the creation of a HHMT, the theoretical and practical workshop, and the monitoring of our internal concordance evaluations ensured the quality of our data over time.

Although the minimum optimal standard of HH performance is unknown, it is clear that a mean compliance of 82% observed in this study is an excellent performance [12]. Of note, during phase 2 the statistical control of our HH process showed “non random variations” (special causes) and this fact is of extraordinary value because when a special cause is noted it should be investigated either to remove it (negative special cause) or to incorporate it (positive special cause). Recently, it has been applied in infection control interventions [28], [29]. In our case, this method has allowed us to detect the influence not only of our intervention (as is shown in the 85% average HH compliance observed in 2011 and in the highest value achieved on world hand hygiene day) but of other “non-intentional” external influences, such as the H1N1 influenza outbreak and the negative influence related to our statutory lay-off proceeding (July-September 2011) which determined a reduction of about 20% of employees. Approximately one a month elapsed since the official announcement of the proceeding until the individual notification to the affected staff. This was a period of obvious anxiety and stress among personnel which we feel could have influenced HH performance. To our knowledge, this is the first study that shows the validity of this methodology in the monitoring of HH process itself and its modulation related to external facts.

Differences according to professional categories, working areas and type of indication have been extensively reported [3], [6], [22], [49], [50]. Unfortunately, poor doctor compliance remains an unsolved and vexing issue [6]. Furthermore, physicians usually are not a role model in HH behaviour, a disappointing conduct that could have a negative influence in other HCWs [22], [50]. Our data, as previously suggested by Pittet el al [51] points out that some differences may have to do with the type of medical speciality, as shown between clinicians and surgeons. Students, irrespective of their professional specialization were better performers. Of note, nursing staff and radiology technicians achieved best results in HH compliance during phase 2. We also have observed, as others [6], [12] a lower compliance in the ED and ICU with respect to conventional wards, which could be related to a higher number of HH opportunities [6]. Notably, phase 2 intervention was especially successful in improving HH performance in these working areas. Regarding the “WHO five moments of hand hygiene”, there was also a higher compliance “after” than “before”, a fact that has been extensively reported [12].

AHRs consumption has usually been considered as a secondary outcome measure to corroborate the results of audit by direct observation [9], [52], [53], [54], [55]. The use of adequate charts for “AHR consumption process” (Poisson charts) showed numerous “positive special causes” during the intervention period. As it has been previously reported, a positive special cause was detected particularly during the 2009 novel H1N1 influenza outbreak [21]. This fact corroborates the validity of our data and clearly illustrates how powerful self-protection is for HCWs [17].

Finally, our data have showed a small but significant decrease in MRSA rate in a low endemic setting through a PEWMA chart (that fits well when very few events are present) despite a significant increase in the use of antibiotics in general and of fluoroquinolones in particular. However, some caution is warranted as to attribute the observed results to the intervention in the absence of a controlled set-up or interrupted time series [56] analysis (which may not be appropriate when there are very few events, as in our case).

### Conclusions

The addition of Continuous Quality Improvement (CQI) methodology may be a key tool for multimodal Hand Hygiene WHO strategy to maintain a good HH performance over time. In addition, the application of Statistical Process Control (SPC) as a time series analysis was shown as a powerful tool that helps us in detecting “non-random” variations (special causes) of the process over time.

### Future research

Future multicenter studies are needed in order to corroborate the external validity of our improvement quality project.

## Supporting Information

Text S1 **Deciding on the Best control chart** (**Statistical Process Control**)**.**
(DOCX)
