# Supplementary material for: “The 3/3 Strategy”: A Successful Multifaceted Hospital Wide Hand Hygiene Intervention Based on WHO and Continuous Quality Improvement Methodology
Source: PLoS One. 2012 Oct 22;7(10):e47200. doi: 10.1371/journal.pone.0047200 (PMC3478274; doi:10.1371/journal.pone.0047200)
Supplement: Text S1 — Deciding on the Best control chart (Statistical Process Control). (DOCX) [file pone.0047200.s001.docx]

**Appendix Text S1.Deciding on the Best control chart (Statistical Process Control).**

**Binomial chart (P-chart ).** “P” stands for either “percent” or “proportion”. The P-chart is the most easily understood and perhaps the most often used control chart. This chart is used frequently in healthcare settings because we track many indicators that look at accuracy, completeness, errors, or the percentage of something done or not done. Because most healthcare indicators that are defined as percentages differ from one time period to another we rarely have equal subgroups when calculating percentages or proportions. Therefore, the p-charts will almost always have stair-step control limits. The mathematic approach is sustained in binomial distribution.

**Poisson Chart (U-chart).** “U” stands for “unequal area of opportunity”. This chart is used frequently in healthcare, especially now that there has been a more concentrated effort to track patient safety indicators. This chart is selected when you conclude that there is not an equal area of opportunity for the defect to occur and the u-chart takes care of this problem by computing a defect rate. The rate use essentially normalizes the differences in denominator size and therefore is applied in epidemiological settings which frequently produces rate-base statistics. The mathematical approach is sustained in Poisson distribution.

**Poisson Exponential Weighted Moving Average control chart (PEWMA).** This is a more sophisticated control chart that shows a weighted average of all past and current observations based on exponentially distribution and perform very well against both, normal and non-normal distributions .The sensitivity and specifity is affected by two main parameters, known as lambda (λ) which usually takes a value from 0.05 to 0.25 and distance from sigma (L) that is usually between 2.6 and 2.8. Published tables helps to select the best parameter combination. This chart has the potential to detect small but sustained increases/decreases of the rates over time.
